# Massive Withdrawal Symptoms and Affective Vulnerability Are Associated with Variants of the *CHRNA4* Gene in a Subgroup of Smokers

**DOI:** 10.1371/journal.pone.0087141

**Published:** 2014-01-30

**Authors:** Judit Lazary, Peter Dome, Iren Csala, Gabor Kovacs, Gabor Faludi, Mari Kaunisto, Balazs Dome

**Affiliations:** 1 Department of Clinical and Theoretical Mental Health, Semmelweis University, Budapest, Hungary; 2 National Institute of Psychiatry and Addictions, Budapest, Hungary; 3 Institute of Behavioral Sciences, Semmelweis University, Budapest, Hungary; 4 Department of Tumor Biology, National Koranyi Institute of Pulmonology, Budapest, Hungary; 5 Institute for Molecular Medicine Finland (FIMM), University of Helsinki, Helsinki, Finland; 6 Folkhälsan Institute of Genetics, Helsinki, Finland; 7 Department of Thoracic Surgery, National Institute of Oncology, Budapest, Hungary; 8 Translational Thoracic Oncology Lab, Department of Thoracic Surgery, Medical University of Vienna, Vienna, Austria; The University of Chicago, United States of America

## Abstract

Heterogeneous phenotypes of complex disorders pose a great challenge for genetic association studies and for the development of personalized treatment strategies. Cluster analysis of phenotypic data has been recently proposed as a reliable auxiliary method for such studies. A cohort of 236 treatment-seeking smokers was investigated after overnight nicotine abstinence. Alpha4 nicotinic acetylcholine receptor (nAChR) subunit-related phenotypes were assessed by the Fagerström Test for Nicotine Dependence (FTND), exhaled carbon monoxide (CO) measurements, the Minnesota Nicotine Withdrawal Scale (MNWS) and the Zung Self-Rating Depression Scale (ZSDS). Seven tag SNPs (single-nucleotide polymorphisms) across *CHRNA4* (the gene encoding alpha4 subunit of the nicotinic acetylcholine receptor) were genotyped and two-step cluster analysis was used for phenotypic cluster characterization. Haplotype estimation was determined by HapStat module of R 2.0 software. Three different phenotypic clusters were identified and the C3 cluster was characterized by the highest ZSDS and MNWS scores compared to others. Furthermore, lifetime prevalence of major depression was significantly higher in the C3 cluster (*p* = 0.019). In genetic association tests, this cluster was also significantly associated with rs3787138 genotypes (*p* = 0.004) while haplotype analyses of three SNPs (rs3787138, rs1044396, rs3787140) revealed that the risk for C3 phenotype was almost three times higher in GCC haplotype carriers compared to others (*p_perm_* = 0.013). This is the first report on a significant association between *CHRNA4* variants and a subgroup of smokers characterized by massive withdrawal symptoms and affective vulnerability. Identification of such a phenotypic cluster can be a pivotal step for further pharmacogenetic studies on ligands of the alpha4 nAChR subunit. Our results suggest that performing cluster analysis in genetic association studies can be proposed for complex disorders.

## Introduction

Nicotine dependence is the most prevalent psychiatric disorder in the world and it is responsible for the highest preventable mortality in developed countries [1], [2]. The increasing body of evidence about the molecular background of smoking behavior suggests that genetic factors have significant role in the pathomechanism but the picture is still not complete. One of the most important candidate genes of smoking behavior is the gene encoding alpha4 subunit of the nicotinic acetylcholine receptor (*CHRNA4*) because among nicotinic acetylcholine receptors (nAChRs) those containing alpha4 subunit have the highest affinity for nicotine, the primary psychoactive component of tobacco [3]. Moreover, the most effective pharmacological agent for smoking cessation, varenicline is a partial agonist on alpha4beta2 nAChRs. Despite of the evident role of *CHRNA4* in multiple smoking-related phenotypes including nicotine dependence, withdrawal and affective symptoms, association studies have provided partly inconsistent results. Positive associations between nicotine dependence and *CHRNA4* were reported in case-control and family studies [4], [5]. Nevertheless, negative findings are also available. In a study of cigarette smokers, nicotine dependence and serum cotinine levels were not associated with *CHRNA4* [6]. Furthermore, Spruell et al. (2012) reported that effect of *CHRNA4* was not significant on smoking cessation outcome in a nicotine replacement therapy (NRT) treatment study [7].

Accumulative data support that depressive phenotypes and smoking have a multifaceted relationship (for a review, see Dome et al. 2010 [8]) and among multiple candidate genes, *CHRNA4* is also implicated in the development of mood disorders. Significant associations between *CHRNA4* and depression and loneliness were demonstrated in a study of elderly population [9]. Accordingly, negative emotionality was proved to be associated with *CHRNA4* [10]. Interestingly, smoking status did not influence these associations between *CHRNA4* and affective phenotype according to these reports [9], [10].

Reconsidering the possible background of these conflicting data on *CHRNA4,* we tried to find an alternative method for detecting significant effect of *CHRNA4* on smoking-related phenotypes. Phenotypic cluster analysis is a recently proposed method for identifying more homogenous subgroups based on parallel analysis of co-existing phenotypic variables [11]. Furthermore, it has been proved to be a promising method for genetic association studies of complex diseases such as asthma and substance use disorders [12]. [13].

Since smoking is a also complex condition, our hypothesis was that cluster analysis can be an appropriate method for finding a subgroup displaying a more serious manifestation with potentially greater load of genetic risk factors. To this end, we have chosen those phenotypic variables that were proved to be linked to *CHRNA4* receptor function. Here we report for the first time a cluster analysis of smoking-related variables and depressive phenotype in an association study of haplotypes within *CHRNA4* in smokers.

## Results

### Descriptive Statistics

The average number of cigarettes per day was 21.2±8.4, breath CO level was 19.0±8.7 and FTND mean score was 6.3±1.2. Mean MNWS score was 12.01±6.1. Mean Zung Self-Rating Depression scale score was 37.7±7.4 in the total population. Comparing different variables between the two genders we found that women scored significantly higher on ZSDS (35.8±6.5 vs. 39.4±8.4, *p* = 0.006) but other factors did not differ from men. Severe depression symptoms (above 48 points on ZSDS) were detected in 7.5% of the sample which is significantly higher than the Hungarian average of current episode of MDD (major depressive disorder) [14]. A comprehensive list of the descriptive variables is presented in Table 1.

**Table 1:** Descriptive characteristics of the study population.

n	236
Age (years; mean±SD)	51.2±12.9
Smoked cigarettes/day	21.2±8.4
FTND score (mean±SD)	6.3±1.2
CO level (ppm) (mean±SD)	19.0±8.7
MNWS score (mean±SD)	12.01±6.1
Heavy smokers (≥20 cigarettes/day)	31.8%
COPD	48.3%
First cigarette under age of 18	43.8%
ZSDS score (mean±SD)	37.7±7.4
ZSDS score above 48	7.5%
Smoking parents	79.7%
Anxiety disorders	10.9%
MDD	9.5%

FTND, Fagerström Test for Nicotine Dependence; MNWS, Minnesota Nicotine Withdrawal Scale; COPD, chronic obstructive pulmonary disorder; ZSDS, Zung Self-Rating Depression Scale; SD, standard deviation; MDD, major depressive disorder.

### Phenotypic Cluster Analysis

**Figure 1:**
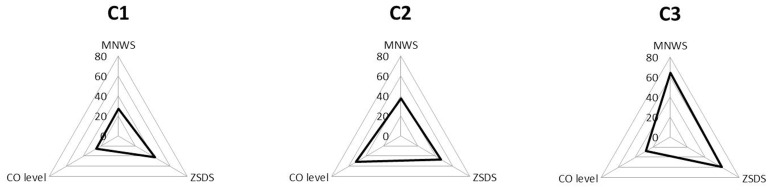
Two-step cluster analysis of three phenotypic variables resulted in three significantly different clusters. Mean scores are represented as percentages of possible maximum points for each measurements. MNWS, Minnesota Nicotine Withdrawal Scale; ZSDS, Zung Self-Rating Depression Scale.

Two-step cluster analysis resulted in the strongest model when MNWS, exhaled CO level and ZSDS score were stepped into the model. In this model 3 different clusters were identified with significantly differing variables (*p*<0.001 for all variables in ANOVA tests). C1 was characterized by the lowest MNWS (8.8±3.8) and ZSDS (34.1±5.1) scores and CO levels (15.0±3.6) (Figure 1, Table 2). Individuals in C2 showed the highest CO levels (30.7±9.3) but they scored lower on MNWS (12.0±4.3) and ZSDS (37.2±5.7) than members of C3 (Figure 1, Table 2). The third cluster was related to the highest MNWS (20.7±3.9) and ZSDS (47.5±6.2) scores but exhaled CO levels were found to be lower (16.5±4.6) than in C2 (Figure 1, Table 2). These results suggest that cluster analysis revealed not only significantly different subgroups but also identified a vulnerable subphenotype of smokers who reacted very sensitively to acute withdrawal of nicotine. Affective hyper-vulnerability as a crucial character of C3 is also demonstrated by the significantly higher lifetime prevalence of MDD in the C3 group than in the others (C1 = 13/113, C2 = 7/48, C3 = 14/48, chi-square = 7.84, df = 2; *p* = 0.019). FTND scores and CPD (cigarettes/day) were also different among the three subgroups identified. Both C2 and C3 had significantly higher FTND score than of C1 (C1 = 6.1±0.9; C2 = 6.6±1.3; C3 = 6.7±1.4; *p_C1/C2_* = 0.023; *p_C1/C3_* = 0.009) but FTND score did not differ between C2 and C3. In line with the highest exhaled CO level in C2, significantly greater amount of CPD was reported in C2 than in C1 (C1 = 19.5±7.5; C2 = 24.5±9.1, C3 = 21.9±8.3; *p_C1/C2_* = 0.001). Gender distribution did not differ significantly among the three clusters.

**Table 2:** MNWS and ZSDS scores and CO levels of the three phenotypic clusters (C1, C2, C3) identified by two-step cluster analysis.

	MNWS (mean±SD)	ZSDS (mean±SD)	CO level (mean±SD)	*p*-value (ANOVA)
**C1** (n = 110)	8.8±3.8	34.1±5.1	15.0±3.6	<0.001
**C2** (n = 47)	12.0±4.3	37.2±5.7	30.7±9.3	<0.001
**C3** (n = 44)	20.7±3.9	47.5±6.2	16.5±4.6	<0.001

MNWS, Minnesota Nicotine Withdrawal Scale; ZSDS, Zung Self-Rating Depression Scale; SD, standard deviation.

### Single Marker Associations

We tested the effects of all SNPs on dependent variables under five models. Single marker association tests provided three nominally significant results: effect of rs6090378 on FTND score (*p* = 0.013); and effects of rs3787138 and rs3787140 on MNWS score variance were significant but not robust enough for Bonferroni corrections (*p* = 0.021; *p* = 0.026, respectively) (Table S1). However, odds ratios for C3 phenotype were significantly higher in G allele carriers of rs3787138 even after Bonferroni corrections (OR = 2.09 (95% CI = 1.0–4.37); *p* = 0.004) while C allele carriers of rs3787140 had almost double the chance to have C3 phenotype with a nominal significance (OR = 1.67 (95% CI = 0.69–4.04); *p* = 0.013). ZSDS, exhaled CO and CPD had no significant relation to SNPs in single marker association models.

### Haplotype Analyzes

Estimation of frequency of haploblock 1 (constructed by rs3787138, rs1044396 and rs3787140, respectively) showed that three haplotypes had greater than 5% frequency in the sample (ATT = 48%; ACT = 38%; GCC = 9%). The frequency of the GCC haplotype was found to be the highest in the C3 cluster (chi-square = 8.68; df = 2; *p* = 0.013, Table 3). First we tested the effect of haplotypes on phenotypic measurements as a continuous variable in the total sample. HapScore test with MNWS score resulted in a marginally significant model (*p_global_* = 0.054). The highest MNWS score was associated with the GCC (*p* = 0.04) and the lowest score with the ATT haplotypes (*p* = 0.02; Table 4). FTND, CPD and ZSDS total scores were not associated with haplotypes in the total sample.

**Table 3:** Frequencies of different haplotypes in phenotypic clusters.

	C1	C2	C3	*p*-value
**ATT**	46%	46%	38%	0.013^a^
**ACT**	41%	38%	31%	
**GCC**	7%	7%	17%	

Allelic components of the presented haplotypes are rs3787138, rs1044396 and rs3787140 SNPs, respectively. ^a^chi-square test indicated a significantly higher frequency of GCC haplotype in C3 compared to non-C3 clusters.

**Table 4:** Haplotypic effect on MNWS score as a continuous variable in GLM and HapScore tests.

	MNWS score				
	GLM			HapScore test^a^	
Haplotypes	Diff.	95%C.I.	*p*-value	score	*p*_effect_
ATT	13.82	ref.		−2.36	0.02
ACT	0.78	−0.49–2.06	0.23	1.04	0.29
GCC	2.44	0.35–4.54	0.02	1.99	0.04

Allelic components of the presented haplotypes are rs3787138, rs1044396 and rs3787140 SNPs, respectively. ^a^ *p_model_* = 0.054; MNWS, Minnesota Nicotine Withdrawal Scale.

**Figure 2:**
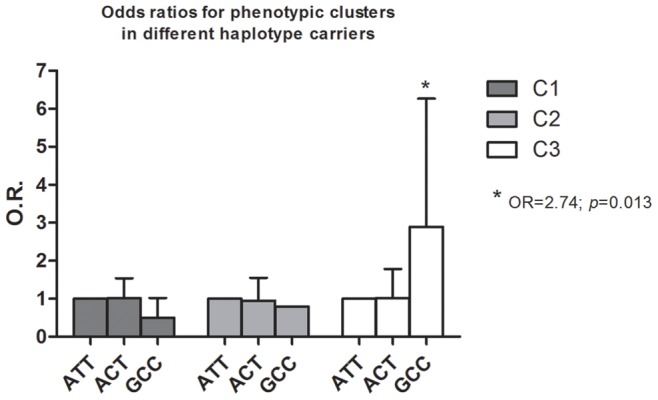
Results of risk analysis of haplotype carrying in different phenotypic clusters. Allelic components of the presented haplotypes are rs3787138, rs1044396 and rs3787140 SNPs, respectively. OR, odds ratio.

In the next step phenotypic clusters were stepped into the model as dependent variables. In HapScore test effect of GCC haplotype was significantly associated with C3 (*p_effect_* = 0.002) and the model was also significant (*p_perm_* = 0.018). Consequently, GLM (general linear model) analysis revealed that odds ratio for having the C3 phenotype was almost three times higher in subjects with GCC haplotype compared to others (OR = 2.74; *p* = 0.013; Table 5; Figure 2).

**Table 5:** Haplotype association tests on C3 phenotypic cluster in GLM and HapScore tests.

	GLM			HapScore Test^a^	
Haplotypes	O.R. for C3	95%C.I.	*p*-value	score	*p*_effect_
ATT	1.0	ref		−1.24	0.21
ACT	1.02	0.62–1.78	0.98	−1.02	0.32
GCC	2.74	1.23–6.09	0.013	2.71	0.002

Allelic components of the presented haplotypes are rs3787138, rs1044396 and rs3787140 SNPs, respectively. ^a^ *p_model_ = 0.018*; GLM, general linear model.

## Discussion

This is the first report demonstrating significant association between haplotypes within *CHRNA4* and phenotypic clusters of smokers. Moreover, despite of the crucial role of alpha4 subunit in the development of nicotine withdrawal symptoms, this is the first study assessing the association between *CHRNA4* variants and acute nicotine withdrawal symptoms.

Our analysis identified a cluster (C3) among smokers which is characterized by significantly more severe withdrawal symptoms with robust affective vulnerability; not only average score on the self-reported depression scale (ZSDS) but also lifetime prevalence of major depression were significantly higher in this subgroup. Of note, the size of this cluster is not negligible: about 20% of the study population belonged to this subgroup. Genotype and haplotype association tests showed that this special phenotype is determined by genetic components: the chance of belonging to the C3 phenotype group was almost three times higher in those carrying the GCC haplotype compared to other haplotype carriers.

The risk haplotype identified in our study has a component described earlier as a risk allele associated with different phenotypes in multiple studies. These findings suggest that C allele carriers of rs1044396 (middle SNP of our haplotype) were more prone to develop nicotine dependence [4]; they have a higher chance to have psychological risk attitude [15] and exhibit weaker attention functions [16]; they are more anxious and emotionally unstable compared to T allele carriers [9], [10] and finally, T allele was found to be protective against nicotine addiction [5]. At the same time, the effect of rs1044396 on response to cessation promoting agents is underinvestigated yet: the only study on this topic failed to find a positive association between this SNP and treatment response in a heterogeneous sample of smokers [7].

The pivotal role of rs1044396 in receptor function was suggested by several authors. Convergent evidence hints that this missense mutation of exon 5 in *CHRNA4* yielded significant increase in acetylcholine sensitivity which can be a reasonable explanation for more serious withdrawal symptoms in carriers of hypersensitive genetic variant of *CHRNA4* in our sample [17], [18]. Regarding the possible role of rs1044396 (also found to be a risk SNP in our study) in receptor sensitivity, Breitling et al. proposed that it is in tight linkage disequilibrium with the functional rs2236196 which is associated with more than two times higher gene expression and greater sensitivity for acute nicotine abstinence compared to its complementary variant [4]. Apart from addictive behaviors, crucial roles of *CHRNA4* exon 5 in emotional processes have also been confirmed [19].

From a pharmacogenetic point of view, it was demonstrated previously that rs2236196 and also rs3787138 (an SNP which was also found to be associated with the C3 cluster in this study) were associated with treatment response to nicotine supplementation therapy and/or varenicline. These results raise the possibility that members of the C3 cluster have altered response to cessation promoting agents which may suggest that smoking cessation studies should not focus on smokers as a whole but rather on phenotypically different subgroups of them [20].

Of note, exhaled CO levels were not the highest in the genetically vulnerable cluster in our cohort. Nevertheless, this finding is partly in line with negative results on the association between serum cotinine levels and *CHRNA4* variants [6]. Our data confirmed previously reported results that controlling for both cigarette experimentation and for quantity smoked during heaviest period of use there are residual influences on nicotine withdrawal (up to 23% of the total variance) [21] suggesting that there may be genetic variance in nicotine withdrawal that is independent of genetic effects on development of nicotine addiction [22].

In previous studies it has been demonstrated that smokers with a history of depression report more severe withdrawal symptoms [23]–[25] and have an increased risk for recurrent episodes of depression after smoking cessation [26]. Based on our data we suggest that this association between nicotine withdrawal and depressive symptoms may not be valid for all smokers generally, it is rather true for a vulnerable cluster which was found to be associated with *CHRNA4* variants.

In conclusion, we identified a clinically remarkable subgroup within smokers characterized by salient withdrawal symptoms and depressive signs. Here we presented a unique approach and studied the effect of *CHRNA4* haplotypes on smoking-related traits using a special phenotypic clustering method. With the help of the phenotypic cluster analysis the possible role of CHRNA4 in smoking behavior and pharmacological actions of cessation agents can be nuanced. Further pharmacological studies are required to extend our knowledge on the role of *CHRNA4* in the treatment of nicotine dependence. Based on these findings we propose cluster analysis combined with haplotype association test as an alternative method for identifying genetically predisposed part of complex disorders and for pharmacogenomic trials.

## Methods

### Ethics Statement

Written informed consent was obtained from all participants and the study was approved by the the Scientific and Research Ethics Committee of the Medical Research Council of Hungary (ad.8-303/2009-1018EKU).

### Subjects and Phenotypic Measurements

A cohort of 350 treatment-seeking smokers from 5 Hungarian Cessation Centers was investigated in the present study. The time between the last smoked cigarette and the clinical interview was not a criterion for study entry, but for the current analysis we selected only those individuals who reported an overnight nicotine abstinence (n = 236). Participants were interviewed by a detailed background questionnaire on smoking-related variables such as quantity and quality of smoking, nicotine content of the cigarette regularly smoked by them and family history of smoking. Nicotine dependence was assessed by the Fagerström Test for Nicotine Dependence (FTND) [27]. The 9-item version of Minnesota Nicotine Withdrawal Scale (MNWS) was used for detection of withdrawal symptoms [28]. The Zung Self-Rating Depression Scale (ZSDS) was performed for estimating depressive phenotype in the previous two weeks [29]. Participants above 3 points of FTND and above 10ppm CO were included in the study. COPD (chronic obstructive pulmonary disease) was validated by spirometry. Clinical interviews, CO measurement and genetic sample collection were performed by clinicians specialized in pulmonology and smoking cessation therapy.

### Genotyping, SNP Selection

**Figure 3:**
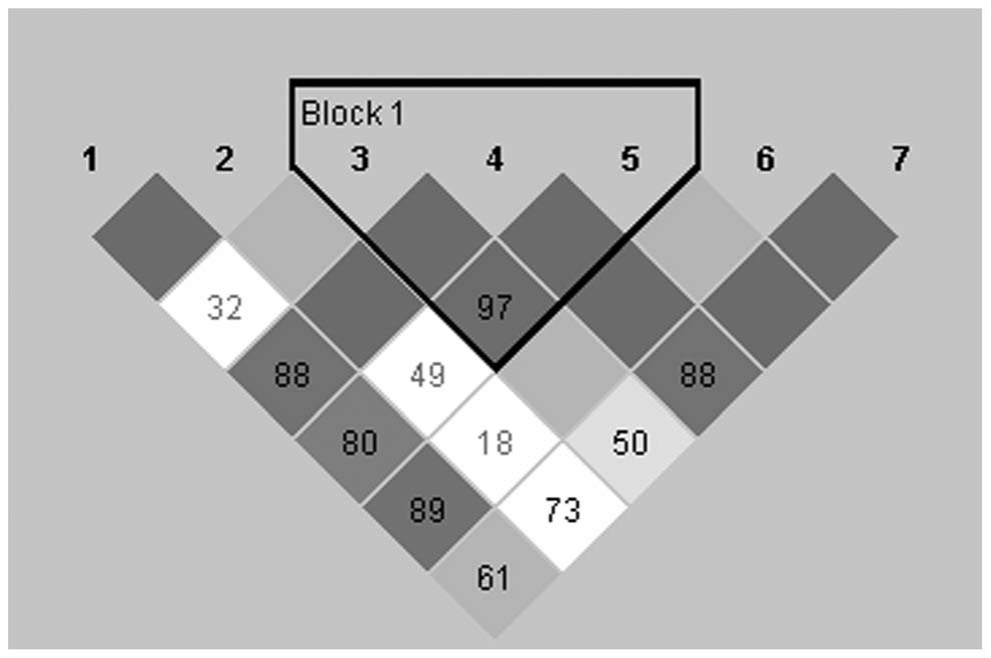
Linkage disequilibrium map of the seven investigated SNPs. 1 = rs4522666, 2 = rs6090378, 3 = rs3787138; 4 = rs1044396; 5 = rs3787140, 6 = rs2093107, 7 = rs755203.

Buccal mucosa samples were taken from all subjects and 7 tag SNPs (rs4522666, rs6090378, rs3787138, rs1044396, rs3787140, rs2093107, rs755203) covering the *CHRNA4* were genotyped with Sequenom MassArray technology and the iPLEX Gold chemistry (Sequenom Inc., San Diego, USA). In this method allele discrimination is based on primer extension with single mass-modified nucleotides followed by MALDI-TOF mass spectrometry. Genotyping was performed by the Technology Centre, Institute for Molecular Medicine Finland (FIMM), University of Helsinki. Genotyping quality was examined by a detailed QC procedure consisting of success rate checks, duplicated samples and positive and negative control samples. Genotyping was done blinded to the phenotypic data. SNPs within *CHRNA4* were selected based on literature and the International HapMap project [4], [5], [10], [30]. Basic characteristics of allelic and genotype distribution were analyzed using Haploview software 4.0 (Table S2) [31]. Minor allele frequencies of each SNPs were not less than 1% in the sample and all genotyped polymorphisms were in Hardy-Weinberg equilibrium. Linkage disequilibrium tests showed that three SNPs (rs3787138 in intron 5, rs1044396 in exon 5 and rs3787140 in intron 2) are in strong linkage disequilibrium (r^2^>95%) constructing one haploblock (Figure 3) according to the criteria of Gabriel et al. (2002) [32].

### Statistical Methods

Two-step cluster method was used for phenotypic cluster analysis based on Ward’s aggregation criterion performed in SPSS 20.0 software. Smoking-related phenotypes, such as FTND score, smoked cigarettes/day (CPD), exhaled CO level, MNWS and ZSDS scores for depressive phenotype were stepped into the model. The strongest model was chosen for genetic association tests (higher than 0.5 of silhouette measure of cohesion and segregation). Haplotype analyses were performed with GLM and HapScore tests under additive model using HapStat modul of R 2.0 software. Rare haplotypes with a frequency less than 5% were excluded from analyses. To avoid false positive results Bonferroni’s correction in single marker association tests and permutation procedures with 1000 random permutations were performed by R 2.0 software similarly to our earlier publication [33]. Permuted p-value less than 0.05 was considered significant in haplotype analyses. Where it was possible, models were adjusted for age and gender (in SMA (standard major axis) and GLM tests).

## Supporting Information

Table S1 Significant results of single marker association tests.
(DOCX)

Table S2 Basic characteristics of the investigated SNPs.
(DOCX)
