# Supplementary material for: Massive Withdrawal Symptoms and Affective Vulnerability Are Associated with Variants of the CHRNA4 Gene in a Subgroup of Smokers
Source: PLoS One. 2014 Jan 30;9(1):e87141. doi: 10.1371/journal.pone.0087141 (PMC3907445; doi:10.1371/journal.pone.0087141)
Supplement: Table S1 — Significant results of single marker association tests. (DOCX) [file pone.0087141.s001.docx]

**Table S1. Significant results of single marker association tests**

| **rs6090378** | **n** | **FTND (mean±SD)** | ***p*-value** |
| --- | --- | --- | --- |
| AA | 203 | 6.3±0.08 | 0.013 |
| GA+GG | 25 | 6.9±0.28 |  |
| **rs3787138** | **n** | **MNWS (mean±SD)** | ***p*-value** |
| AA | 149 | 11.6±0.48 | 0.021 |
| AG | 49 | 12.6±0.92 |  |
| GG | 3 | 22.3±2.33 |  |
| **rs3787140** | **n** | **MNWS (mean±SD)** | ***p*-value** |
| TT | 166 | 11.7±0.47 | 0.026 |
| TC | 32 | 12.4±1.03 |  |
| CC | 3 | 22.3±2.33 |  |

MNWS, Minnesota Nicotine Withdrawal Scale; ZSDS, Zung Self-Rating Depression Scale; SD, standard deviation
