# Supplementary material for: Massive Withdrawal Symptoms and Affective Vulnerability Are Associated with Variants of the CHRNA4 Gene in a Subgroup of Smokers
Source: PLoS One. 2014 Jan 30;9(1):e87141. doi: 10.1371/journal.pone.0087141 (PMC3907445; doi:10.1371/journal.pone.0087141)
Supplement: Table S2 — Basic characteristics of the investigated SNPs. (DOCX) [file pone.0087141.s002.docx]

**Table S2. Basic characteristics of the investigated SNPs**

|  | **Alleles** | **MAF** | **% Geno** | **Location** |
| --- | --- | --- | --- | --- |
| rs4522666 | A/G | 0.38 | 99.6 | 3’UTR |
| rs6090378 | A/G | 0.06 | 100 | Exon 6 |
| rs3787138 | A/G | 0.14 | 100 | Intron 5 |
| rs1044396 | C/T | 0.48 | 98.3 | Exon 5 |
| rs3787140 | T/C | 0.09 | 99.6 | Intron 2 |
| rs2093107 | G/A | 0.08 | 100 | promoter |
| rs755203 | C/T | 0.50 | 99.6 | promoter |

MAF, minimal allele frequency
